# Depth‐Wise Changes in Tibial and Femoral Human Knee Joint Cartilage at Different Severities of Osteoarthritis

**DOI:** 10.1002/jor.70104

**Published:** 2025-12-13

**Authors:** Katja Honkapää, Atte S.A. Eskelinen, Santtu Mikkonen, Mohammadhossein Ebrahimi, Mikko A.J. Finnilä, Simo Saarakkala, Rami K. Korhonen, Martin Englund, Petri Tanska

**Affiliations:** ^1^ Department of Technical Physics University of Eastern Finland Kuopio Finland; ^2^ Department of Environmental and Biological sciences University of Eastern Finland Kuopio Finland; ^3^ Research Unit of Health Sciences and Technology, Faculty of Medicine University of Oulu Oulu Finland; ^4^ Biocenter Oulu University of Oulu Oulu Finland; ^5^ Department of Diagnostic Radiology Oulu University Hospital Oulu Finland; ^6^ Faculty of Medicine, Department of Clinical Sciences Lund, Orthopaedics, Clinical Epidemiology Unit Lund University Lund Sweden

**Keywords:** articular cartilage, collagen, knee joint, osteoarthritis, proteoglycans

## Abstract

Articular cartilage undergoes structural and compositional changes during osteoarthritis (OA), one of the most common joint diseases. Earlier research shows that these changes are dependent on species and joint site, and they also vary across cartilage depth. In this study, we analyzed the depth‐wise proteoglycan and collagen contents, as well as collagen fibril orientation angle and fibril alignment in human tibial and femoral cartilage at different severities of osteoarthritis. Samples were divided into normal cartilage, moderate OA cartilage, and severe OA cartilage based on OARSI grade. Consistent with earlier research, proteoglycan and collagen contents were generally lower in samples of greater OA severity, with the notable exception of a higher collagen content in femoral severe OA cartilage. Femoral severe OA cartilage had considerably lower proteoglycan content than femoral normal or moderate OA cartilage, and its collagen fibril orientation and anisotropy became more uniform throughout cartilage thickness. Qualitative analysis between tibial and femoral cartilage sites also revealed a gradual progressive structural and compositional degradation in tibial cartilage compared to femoral cartilage, in which the structure and composition remained relatively unchanged until the severe OA severity. With this depth‐wise and site‐specific compositional and structural information, our work elucidates disease progression in human cartilage.

## 1 Introduction

Osteoarthritis (OA) is a common joint disease that includes deterioration of articular cartilage and functional impairment of various joint tissues [1]. Articular cartilage, the tissue that covers articulating surfaces in synovial joints, dissipates loads and provides smooth joint movement [2]. The main constituents of cartilage are collagens (mainly type II), proteoglycans (PGs), and interstitial fluid, in addition to chondrocytes, the only cell type in the tissue. Noncalcified articular cartilage has traditionally been divided depth‐wise into three zones according to collagen orientation: superficial (collagen fibers parallel to cartilage surface), middle (fibers oriented randomly), and deep (fibers perpendicular to cartilage surface) [3]. Articular cartilage attaches to the underlying bone through calcified cartilage, and a tidemark can be seen between noncalcified and calcified cartilage [2].

In articular cartilage, fluid fraction and densities of chondrocytes, collagen, and PGs vary in a zone‐dependent manner [4, 5, 6]. Moreover, previous animal studies [7, 8, 9, 10, 11] and a few on humans [12, 13, 14, 15, 16, 17, 18] have reported compositional and biomechanical differences in cartilage between joint sites, possibly due to site‐specific loading environment (e.g., amplitudes and rates) [19]. As OA is most prevalent in the knee joint, tibial and femoral articular cartilage sites have been among the most investigated [20]. In cartilage, OA progression is characterized by disorganization of the collagen fiber network [21, 22], PG loss [23, 24], and an increase in fluid content [23, 25], ultimately manifesting in a deterioration of the mechanical function of the tissue [15, 16]. Yet, research is scarce on how depth‐wise structure and composition of human articular cartilage are altered at femoral and tibial joint sites. This information is crucial for understanding how OA progresses in different sites of human joint and is a prerequisite for the development of timely interventions to halt OA progression.

In this study, we added new data to and reanalyzed a previously published data set [14, 16] to characterize depth‐wise changes in human tibial and femoral cartilage at different severities of OA. We specifically investigated PG content and collagen content, orientation, and anisotropy. Additionally, we conducted a secondary analysis by comparing these depth‐wise changes qualitatively between the tibial and femoral cartilage sites.

## 2 Methods

### 2.1 Sample Preparation and Measurements

We added new data to and reanalyzed a previously published data set [14, 16]. Human tissue was acquired with informed consent from donors or their relatives. The studies were approved by the National Authority for Medicolegal Affairs and the ethical committee of North Savo Hospital District (Ethical Permission Number 134/13.02.00/2015) and the regional ethical review board of Lund University (Dnr 2015/39 and Dnr 2016/865), respectively. A brief description is provided below.

**Figure 1:**
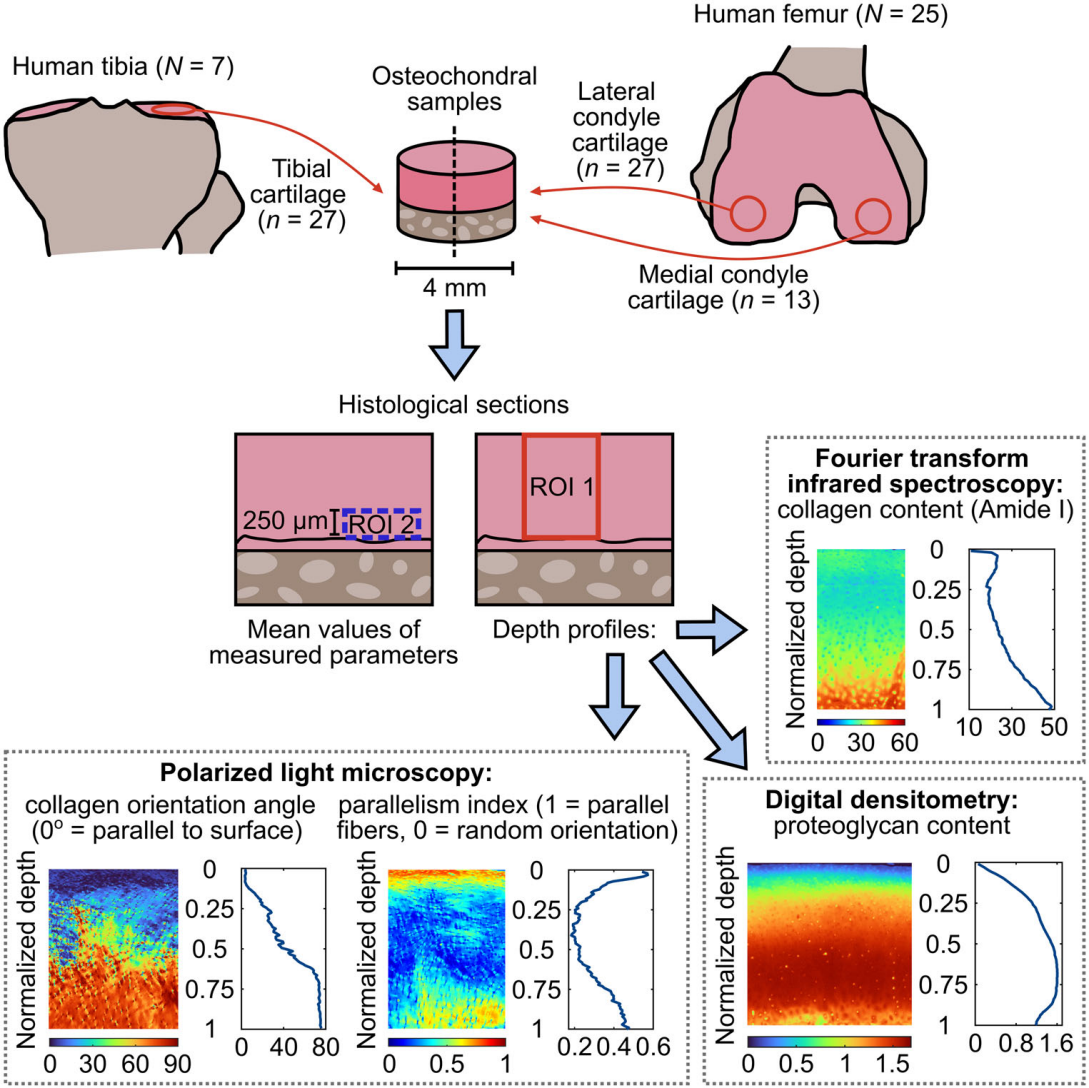
Overview of the study. The regions of interest (ROIs) were defined from cartilage surface to the tidemark (ROI 1) and from the tidemark to 250 µm above it (ROI 2).

We measured 27 human osteochondral samples from tibial cartilage and 40 from femoral cartilage (diameter = 4 mm, Table 1). Tibial samples originated from seven donors (1 woman and 6 men aged 68–79 years, mean age 71.4 ± 5.2 years). Femoral samples originated from 15 total knee replacement patients (8 women and 7 men aged 50–79 years, mean age 65.0 ± 7.3 years) and 10 donors (5 women and 5 men aged 18–77 years, mean age 53.4 ± 17.8 years). Fresh samples were stored in phosphate buffered saline with enzymatic inhibitors (pH 7.4). After fixation, decalcification, and dehydration, we prepared Safranin‐O‐stained and ‐unstained histological sections (Figure 1).

**Table 1:** Cartilage sample distribution by OARSI grade.

	Normal cartilage		Moderate OA		Severe OA		
	OARSI 0	OARSI 1	OARSI 2	OARSI 3	OARSI 4	OARSI 5	Total
Medial tibial	*n* = 0	*n* = 0	*n* = 3	*n* = 0	*n* = 8	*n* = 0	*n* = 11
Lateral tibial	*n* = 2	*n* = 3	*n* = 2	*n* = 2	*n* = 7	*n* = 0	*n* = 16
Medial femoral	*n* = 1	*n* = 4	*n* = 5	*n* = 0	*n* = 3	*n* = 0	*n* = 13
Lateral femoral	*n* = 4	*n* = 10	*n* = 7	*n* = 3	*n* = 1	*n* = 2	*n* = 27

We determined the OA severity of each sample using Safranin‐O‐stained histological sections. Three independent experts graded the samples with OARSI grading system [26] and the samples were pooled into a normal cartilage group (OARSI grade 0–1.5), a moderate OA cartilage group (OARSI grade 2–3.5), and a severe OA cartilage group (OARSI grade 4–5.5).

We used digital densitometry (DD) of Safranin‐O‐stained sections to determine PG content. The DD measurements were conducted with a conventional light microscope (Nikon Microphot FXA, Nikon Co., Tokyo, Japan, pixel size = 3.09 × 3.09 µm), a CCD camera, and a band‐pass light filter (λ = 492 ± 5 nm) [27]. Three 3‐µm‐thick sections were analyzed per sample.

Unstained sections were imaged using Fourier‐transform infrared microspectroscopy (FTIR) to determine collagen content (Amide I, wavenumber range 1720–1595 cm^−1^). We conducted the FTIR measurements using a spectrometer (Agilent Cary 600, Agilent Technologies Inc., Santa Clara, CA, USA) with a spectral resolution of 4 cm^−1^ and a spatial resolution of 5.5 × 5.5 µm [27]. Three 5‐µm‐thick sections were analyzed per sample.

Additional unstained sections were imaged using polarized light microscopy (PLM) to determine collagen orientation angle and parallelism index (PI). The collagen orientation angle is based on Stokes parameters (0° = parallel to tissue surface, 90° = perpendicular to surface), and parallelism index depicts collagen anisotropy ranging from 0 (isotropic = fibers randomly oriented) to 1 (anisotropic = fibers parallel to each other) [28]. The collagen orientation angle measurements were conducted with Abrio PLM system (CRi, Inc., Woburn, MA, USA) with an image pixel size of 2.53 × 2.53 µm [29]. The anisotropy measurements were conducted with another custom‐made PLM system consisting of a light microscope (Leitz Ortholux II POL, Leitz Wetzlar, Wetzlar, Germany), band‐pass light filter (λ = 630 ± 30 nm, Edmund Optics Inc., Barrington, NJ, USA), monochrome CMOS camera (sensor pixel size: 3.45 μm, BFS‐U3‐88S6M‐C FLIR Blackfly® S, FLIR Systems Inc., Wilsonville, OR, USA), and a 2.5× magnification lens resulting in an image pixel size of 1.4 × 1.4 µm [30]. Three 5‐µm‐thick sections were analyzed per sample.

### 2.2 Data Analysis

We conducted depth‐wise analysis for PG and collagen content, as well as collagen fibril orientation angle and parallelism index data. We defined rectangular regions of interest from the cartilage surface to the tidemark and averaged these values along the transverse (horizontal) direction using a custom‐made Matlab code (r2023b, MathWorks Inc., MA, USA) to create a single profile for each section. The width of this ROI (ROI 1 in Figure 1) was 2.0 mm in DD images, 1.1 mm in FTIR images, and 1.5 mm in PLM images. Lastly, each profile was normalized across cartilage depth (by linear interpolation to 100 points) so that 0 = surface and 1 = tidemark, and the average profile for each sample was calculated (average from the three sections).

In later stages of OA, the superficial zone of cartilage is partially eroded. Therefore, the depth‐wise comparison between different OA severities becomes complicated [31]. To ensure that our observations are not influenced by potential superficial tissue erosion and thickness variations in moderate OA, we calculated the average values of these structural and compositional parameters from the separate ROI located near the tidemark. These (non‐normalized) ROIs extended 250 µm into the deep zone from the tidemark (ROI 2 in Figure 1) and were used to acquire averaged values for the deep zone cartilage in each sample (average from the three sections).

### 2.3 Statistical Analysis

**Figure 2:**
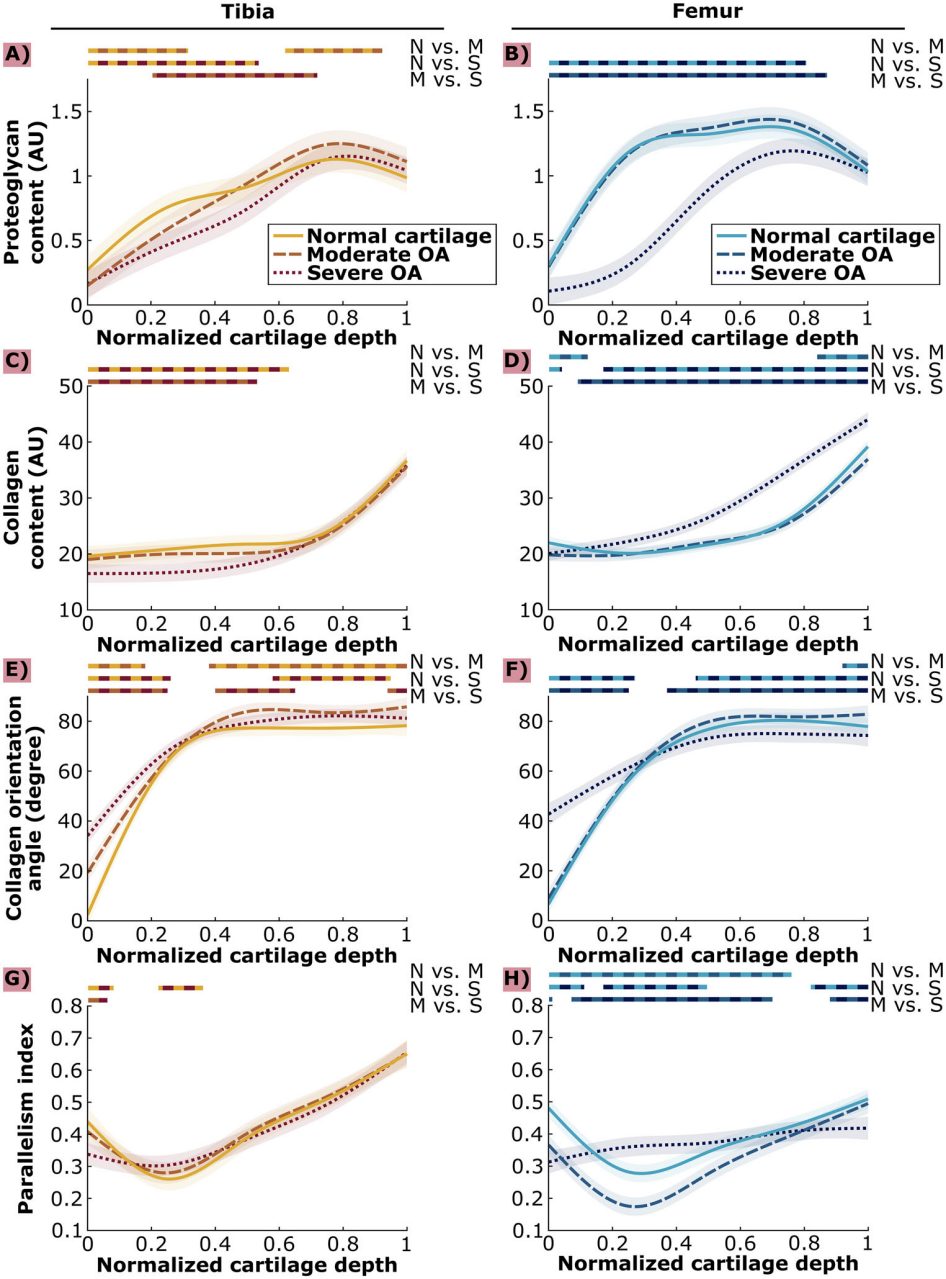
Proteoglycan content (A–B), collagen content (C–D), collagen orientation angle (E–F), and parallelism index (G–H) values for normal cartilage (OARSI 0–1.5), moderate OA cartilage (OARSI 2–3.5) and severe OA cartilage (OARSI 4–5.5) across normalized cartilage depth for tibial cartilage (A, C, E, G) and femoral cartilage (B, C, F, H). Lines: estimated means, shading: ±95% confidence interval, color bars: depths at which statistically significant difference was observed between OA severity groups (N: normal, M: moderate OA, S: severe OA).

We used a linear mixed effects model [32] to account for the sample interdependencies arising from shared patient or donor origins. In the model, the interaction of the OARSI group and the normalized cartilage depth was considered a fixed effect, and the subject was accounted with a random intercept and variance components structure. The normalized depth was modeled with a cubic spline with a maximum of three knots. For the deep zone averages (ROI 2, Figure 1), we used otherwise similar linear mixed model, but the OARSI group was considered as a fixed effect. In our preliminary analysis, we also checked the significance of the medial/lateral compartment (fixed effect); see the Supporting Material for details (Table S1). Statistical analysis was conducted with RStudio (2024.12.1, Posit PBC, MA, USA) applying R version 4.5.0 [33]. False discovery rate was tested for multiple testing. Depth‐wise profile figures in Figure 2 show the linear mixed effects model‐estimated group means with 95% confidence intervals. Statistically significant differences were determined by examining confidence intervals.

## 3 Results

### 3.1 Proteoglycan Content

In tibia, the PG content in *moderate OA* cartilage was significantly lower in the superficial–middle tissue regions and higher in deep tissue regions than in *normal* cartilage (normalized thicknesses 0%–34% and 67%–100%, respectively, Figure 2A). The PG content in *severe OA* cartilage was significantly smaller than in *normal* cartilage in the superficial‐deep tissue regions (normalized thickness 1%–58%), and significantly smaller than in *moderate OA* cartilage in the middle–deep tissue regions (normalized thickness 22%–78%).

In femur, the PG content in *severe OA* cartilage was significantly smaller than in *normal* and *moderate OA* cartilage almost throughout the tissue depth (normalized thicknesses 0%–87% and 0%–94%, respectively, Figure 2B).

**Figure 3:**
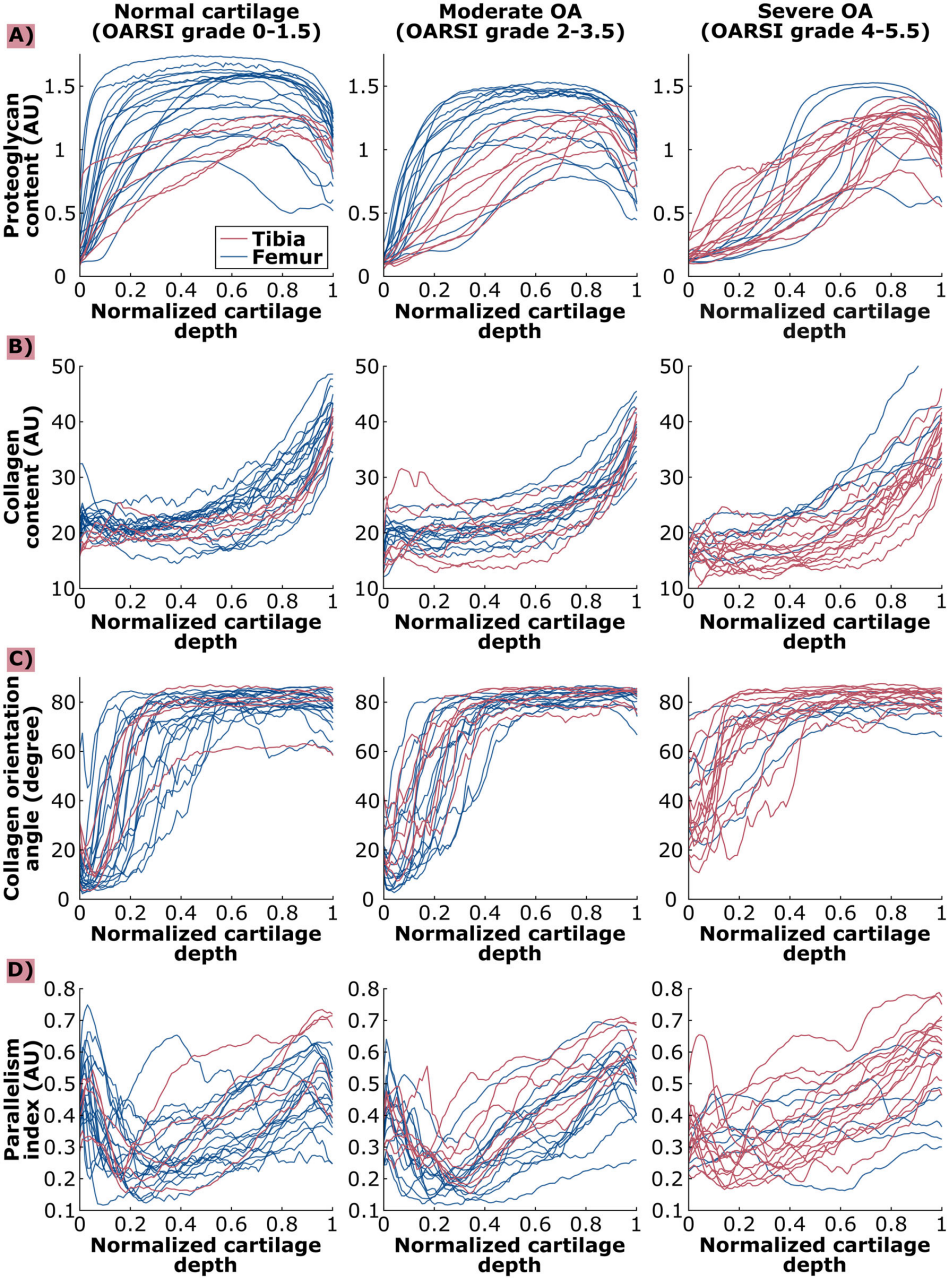
Proteoglycan content (A), collagen content (B), collagen orientation angle (C), and parallelism index (D) values for normal cartilage (OARSI 0–1.5), moderate OA cartilage (OARSI 2–3.5) and severe OA cartilage (OARSI 4–5.5) across normalized cartilage depth for tibial cartilage and femoral cartilage. Lines: individual profiles.

Femoral cartilage had qualitatively higher PG content compared to tibial (Figure 3A) in the middle regions of *normal* and *moderate OA* cartilage. However, we observed large sample‐to‐sample variation. The PG content profiles were similar between tibial and femoral cartilage throughout the depth in later severities of OA.

### 3.2 Collagen Content

In tibia, the collagen content in *severe OA* cartilage was significantly lower than in *normal* and *moderate OA* cartilage in the superficial–middle tissue regions (normalized thicknesses 0%–63% and 0%–53%, respectively, Figure 2C).

In femur, the collagen content in *moderate OA* cartilage was significantly lower than in *normal* cartilage in the superficial and deep tissue regions (normalized thicknesses 0%–12% and 84%–100%, Figure 2D). The collagen content in *severe OA* cartilage was significantly smaller than in *normal* cartilage in superficial tissue regions (normalized thickness 0%–4%). In deeper tissue, the collagen content in *severe OA* cartilage was significantly higher than in *normal* or *moderate OA* cartilage (normalized thicknesses 17%–100% and 9%–100%, respectively).

We observed generally higher collagen content values in femoral cartilage compared to tibial cartilage (Figure 3B). In *normal* cartilage, collagen content was qualitatively higher in femoral cartilage than in tibial cartilage in the superficial and deep tissue regions. In addition, *severe OA* group′s collagen content was qualitatively higher in the deep tissue region of femoral cartilage compared to tibial cartilage.

### 3.3 Collagen Orientation Angle

In tibia, the collagen orientation angle increased (fibers more perpendicular to the surface) with OA severity in the superficial–middle tissue regions (Figure 2E). Specifically, the orientation angle was significantly higher in *moderate OA* cartilage than in *normal* cartilage (normalized thickness 0%–18%), and significantly higher in *severe OA* than in both *moderate OA* and *normal* cartilage (normalized thickness 0%–26% and 0%–25%, respectively). In deeper tissue regions, the collagen orientation angle was significantly higher in *moderate OA* cartilage compared to *normal* cartilage (normalized thickness 38%–100%). The collagen orientation angle in *severe OA* cartilage was significantly higher than in *normal* cartilage in the deep tissue region (normalized thickness 58%–100%), while it was significantly lower compared to *moderate OA* cartilage (normalized thicknesses 40%–65% and 94%–100%).

In femur, the collagen orientation angle was significantly higher only in *severe OA* cartilage compared to *normal* and *moderate OA* cartilage in the superficial‐middle tissue regions (normalized thicknesses 0%–27% and 0%–25%, respectively, Figure 2F). In deeper tissue, the collagen orientation angle was significantly lower in *severe OA* cartilage than in both *moderate OA* and *normal* cartilage (normalized thicknesses 37%–100% and 46%–100%, respectively). In addition, *moderate OA* cartilage had a significantly higher collagen orientation angle than *normal* cartilage close to the tidemark (normalized thickness 92%–100%).

The collagen orientation angles were similar between tibial and femoral cartilage, except for *severe OA* cartilage in the deeper tissue regions, where femoral cartilage had qualitatively lower values than tibial cartilage (Figure 3C).

### 3.4 Collagen Parallelism Index

In tibia, cartilage PI values showed very localized changes with OA severity (Figure 2G). *Severe OA* cartilage had significantly smaller PI (more randomly oriented fibrils) compared to *moderate OA* and *normal* cartilage in the superficial tissue region (normalized thicknesses 0%–6% and 0%–8%, respectively), while the PI of *severe OA* cartilage was significantly larger (more parallel fibrils) compared to *normal* cartilage in the middle tissue region (normalized thickness 22%–36%).

In femur, the PI curve of *severe OA* cartilage was almost uniform (range of PI values 0.31–0.42) along tissue depth compared to *moderate OA* or *normal* cartilage (ranges 0.17–0.49 and 0.28–0.51), i.e., the zonal‐ and depth‐dependency in the fibril parallelism was lost in *severe OA* (Figure 2H). *Severe OA* cartilage had significantly smaller PI compared to *moderate OA* and *normal* cartilage in superficial and deep tissue regions (normalized thicknesses 0%–1% and 88%–100% for *moderate OA*, and 0%–11% and 88%–100% for *normal* cartilage). In turn, *severe OA* cartilage had significantly larger PI in the middle–upper deep tissue regions compared to *moderate OA* and *normal* cartilage (normalized thicknesses 7%–70% and 17%–50%, respectively). *Moderate OA* and *normal* cartilage exhibited the typical zonal‐ and depth‐dependent shape in the PI curve [34, 35, 36], and *moderate OA* cartilage had significantly smaller PI than *normal* cartilage in superficial to deep tissue regions (normalized thickness 0%–76%).

PI values showed larger changes in femoral than tibial cartilage (Figure 2G–H). Tibial cartilage had qualitatively higher PI values compared to femoral cartilage in the deep tissue regions of cartilage in all OA severities (Figure 3D).

### 3.5 Deep Zone ROI Analysis

**Figure 4:**
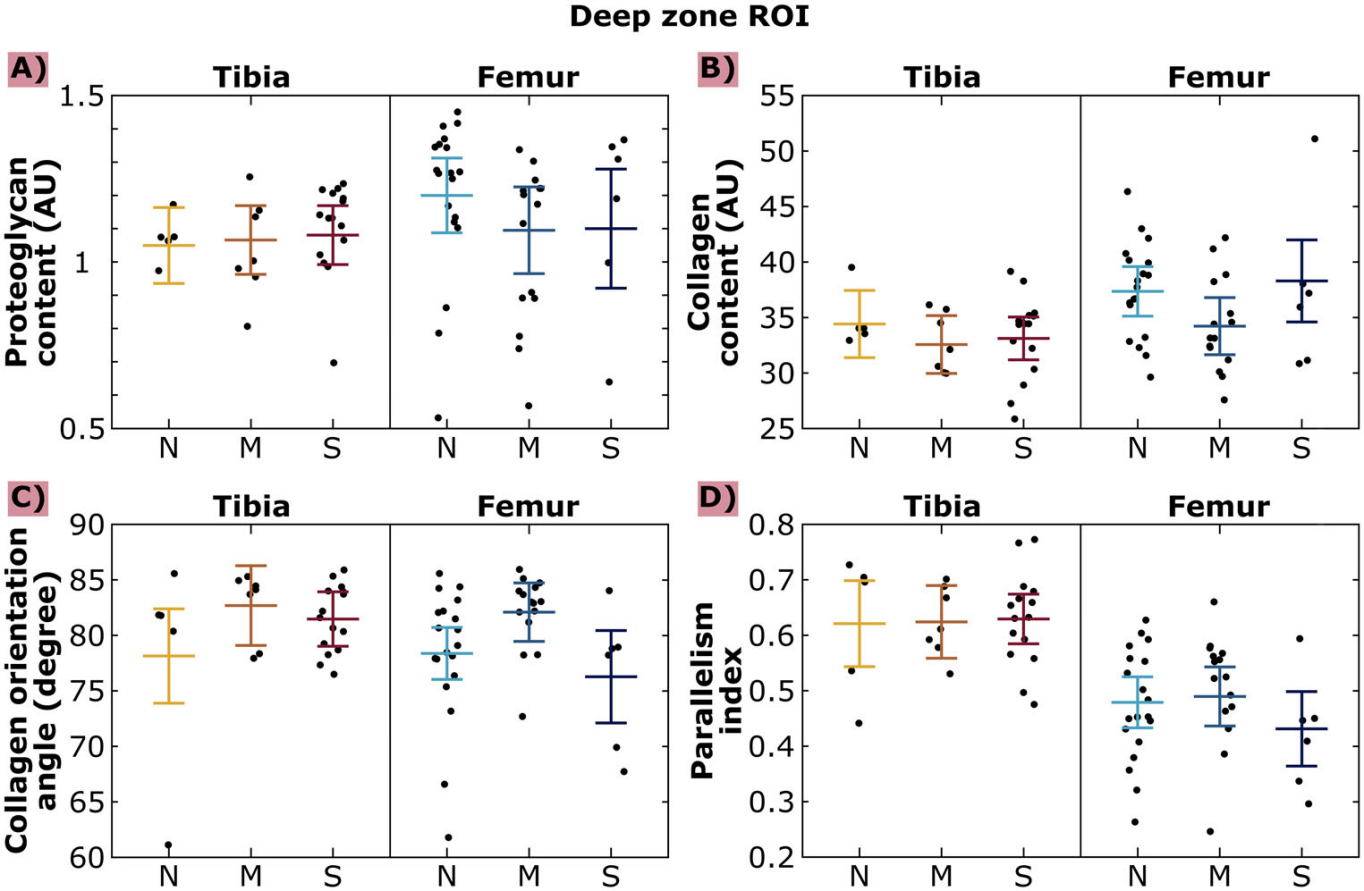
Proteoglycan content (A), collagen content (B), collagen orientation angle (C), and parallelism index (D) from 250 µm thick regions of interest (defined from the tidemark towards superficial cartilage) in the deep zone of tibial and femoral cartilage in cases of normal cartilage (OARSI 0–1.5), moderate OA cartilage (OARSI 2–3.5) and severe OA cartilage (OARSI 4–5.5). N: normal, M: moderate OA, S: severe OA. The colored bars represent the estimated means and ±95% confidence intervals, and black dots show the individual data points. No statistically significant differences between OA severities were found.

In the deep zone ROI analysis (ROI 2 in Figure 1), we did not observe any statistically significant differences in tibial or femoral cartilage PG content, collagen content, collagen orientation, or PI between the OA groups (Figure 4A–D). Qualitatively, we observed higher PI values in tibial than femoral cartilage (Figure 4D).

## 4 Discussion

In this study, we remeasured, reanalyzed, and compared the composition and structure of human tibial and femoral cartilage at different severities of osteoarthritis. We conducted depth‐wise analysis for proteoglycan content, collagen content, collagen orientation, and collagen anisotropy (Figure 2). In general, increasing OA severity was linked to lower proteoglycan and collagen content. Two exceptions to this general finding were observed: 1) for tibial *moderate OA* cartilage in the deep zone, where PG content was higher than in *normal* tibial cartilage, and 2) for femoral *severe OA* cartilage, which had larger collagen content in deep tissue compared to femoral *normal* and *moderate OA* cartilage. The largest changes in collagen orientation angle were found in the superficial tissue; fibrils were less parallel to surface in both tibial and femoral *severe OA* cartilage compared to *normal* and *moderate OA* cartilage. Collagen anisotropy followed a typical zonal‐ and depth‐dependent parabolic curve shape with depth‐wise localized changes in anisotropy values in tibial cartilage across different severities of OA, while in femoral cartilage, this typical curve shape was lost and became more uniform in *severe OA*. These novel findings provide new insights into depth‐wise, site‐specific compositional and structural changes in different OA severities, which are specifically important for understanding OA progression in humans.

Earlier research on human articular cartilage has shown a decrease in PG content [23, 24, 37, 38, 39] and collagen content [23, 37] when OA progresses. Our results showed a gradual decrease of PG content in the superficial–middle tissue region of tibial cartilage with advancing disease severity, with a more pronounced decrease of PG content in femoral *severe OA* cartilage (Figure 2B) and of collagen content in tibial *severe OA* cartilage (Figure 2C). Hanifi et al. found no PG or collagen content changes in tibial *moderate OA* cartilage, but in their analysis content variation along the tissue thickness was not accounted for [31]. Kumar et al. also found the PG content decrease in femoral cartilage to be statistically significant only in *severe OA* (OARSI grade > 3) [38], as compared to earlier disease. Our results are in line with previous findings and are conducted here with a consistent analysis between sites.

Earlier human cartilage research has also noted a more random fibril orientation [22, 23, 31, 39] when OA progresses. Bi et al. found that the orientation of collagen fibrils changed in the superficial tissue in tibial *early OA* cartilage and throughout the tissue depth in tibial *severe OA* cartilage [39]. Our results agree with increasing collagen orientation angle (less parallel to surface) in the superficial–middle tissue region of tibial cartilage as OA progresses. In femoral cartilage, this increase was only seen in *severe OA* cartilage. Szarek et al. found a higher dispersion of collagen fibers in the superficial and deep zones as well as lower dispersion in the middle zone of femoral *moderate OA* compared to *healthy* cartilage [22]. This result is similar to the PI profiles we measured from *severe OA* cartilage, especially in femoral cartilage (lower PI in superficial and deep tissue, higher in middle as compared to *normal* cartilage, Figure 2H). Hanifi et al. measured a more random fibril orientation in tibial *moderate OA* cartilage than in OARSI 1 grade cartilage [31]. Consistent with this, our femur PI profiles show that anisotropy decreased in *moderate OA* cartilage compared to *normal* cartilage. In addition, the *severe OA* collagen orientation angle profiles of both tibial and femoral cartilage show a more uniform angle distribution (e.g., range of angle values in femur 43–75 degrees) compared to the *moderate OA* ones (in femur 9–83 degrees).

Although cartilage thicknesses in different OA groups were similar in the current data set [13, 15], visual observation showed significant tissue erosion in some of the samples with the highest OARSI grades. We omitted those samples from the analysis in this paper, as they did not provide meaningful depth‐wise profiles. Additionally, cartilage swelling may compensate for thickness losses in degenerated tissue in *moderate OA*. To add analysis independent of surface tissue erosion, we also investigated deep zone changes (ROI 2, Figure 1).

We did not find any statistically significant differences in the deep zone ROI comparison (Figure 4). Based on our results, deep tissue seems to preserve its structure and composition well during OA progression. The lack of differences indicates that the degradation of the superficial zone may have affected the differences seen in the depth‐wise analysis (Figure 2) where values were normalized to cartilage depth. These differences were seen between *normal* and *moderate OA* tibial cartilage PG content, between *normal* and *moderate OA* tibial cartilage collagen orientation angle, between *moderate OA* and *severe OA* tibial cartilage collagen orientation angle, between the collagen content and collagen orientation angle of all femoral OA groups, between *normal* and *severe OA* femoral cartilage PI, and between *moderate OA* and *severe OA* femoral cartilage PI. Lack of deep tissue changes during OA progression can be expected, as the degradation typically starts from the cartilage surface or manifests in osteophytes and is seen in deep cartilage only in late disease [21, 29, 39]. Earlier research has also shown lack of changes in deepest parts of tissue [29, 40].

Comparing tibial and femoral cartilage PG content qualitatively (Figure 3A), the PG content was higher in femoral than tibial cartilage in *normal* and *moderate OA* cartilage, but not in *severe OA* cartilage. We also measured higher collagen content values in the superficial and deep tissue regions of *normal* femoral cartilage and in the deep tissue region of *severe OA* femoral cartilage compared to tibial cartilage. These findings suggest that femoral cartilage is more resistant to OA‐like compositional changes (PG and collagen loss). This is supported by a study by Linus et al. who found changes in the mechanical properties of tibial cartilage earlier during OA progression compared to femoral cartilage [17]. Lahm et al. found that collagen and PG contents were higher in healthy femoral cartilage compared to healthy tibial cartilage, with this site‐specific difference decreasing with OA progression [37].

In our results, earlier degradation of tibial cartilage compared to femoral cartilage could be seen in the superficial collagen orientation angles (Figure 2E). Contrary to this, we found a decrease in superficial femoral cartilage collagen content already in *moderate OA*, although this was depth‐wise very localized. In addition, changes in PI happened at an earlier disease severity in femoral cartilage than in tibial cartilage. Taken together, *severe OA* femoral cartilage values were highly different from other femoral OA disease groups. Thus, OA changes could generally be seen earlier in tibial cartilage than femoral, but changes in femoral cartilage became notably strong in *severe OA* cartilage.

Generally, cartilage tissue and its components erode in OA, which is why our observation of increasing PG content in the deep tissue regions of tibial cartilage and increasing collagen content in middle‐deep tissue regions of femoral cartilage raises interest. Because our study was not longitudinal, individual differences are possible. However, PG content increase in *moderate OA* cartilage may be due to increased PG synthesis in chondrocytes [41]. Some earlier studies do also note slight increases in the collagen content of some samples [24, 29, 37]. Mäkelä et al. found increased collagen content in rabbit OA model femoral cartilage [9]. Our collagen content analysis measured absorbance in the Amide I region, which also corresponds to collagen type I. If the collagen content alters to consist more largely of collagen type I during osteoarthritis [42], the mechanical function of cartilage deteriorates even if the collagen content stays high.

This study has some limitations. First, our sample sizes were small in some comparison groups. Second, the tibial and femoral samples originated from different subjects, making them not directly comparable. For this reason, we kept our analysis between tibia and femur qualitative. Earlier research has noted differences in the cartilage compositions of the lateral and medial sides of the same location (tibia/femur), e.g., higher collagen content in lateral than medial tibia [10, 11, 37]. Our statistical analysis showed the joint compartment to be a significant factor, but we pooled the lateral and medial side samples because of our already‐limited sample size. The *normal* tibial cartilage samples were all from lateral compartments (Table 1). The lateral/medial compartment analysis can be seen in the Supporting Material (Table S1).

In conclusion, we compared depth‐wise proteoglycan content, collagen content, collagen orientation, and collagen anisotropy of human tibial and femoral cartilage at different severities of OA. The strength of our work is the consistent depth‐wise analysis methodology between different sites. Overall, proteoglycan and collagen contents were generally lower in samples of greater OA severity, with the notable exception of higher collagen content in femoral *severe OA* cartilage. We also found a gradual progressive structural and compositional degradation of tibial cartilage compared to femoral cartilage during OA progression, while the structure and composition in femoral cartilage remained relatively unchanged until the *severe OA* severity. These compositional characteristics of human articular cartilage help to understand the disease progression in different anatomical sites.

## Author Contributions

**Katja Honkapää:** research design, analysis, interpretation, manuscript drafting and revision. **Atte S. A. Eskelinen:** research design, interpretation, manuscript revision. **Santtu Mikkonen:** research design, analysis, interpretation, manuscript revision. **Mohammadhossein Ebrahimi:** research design, acquisition, analysis, interpretation, manuscript revision. **Mikko A. J. Finnilä:** research design, interpretation, manuscript revision. **Simo Saarakkala:** research design, interpretation, manuscript revision. **Rami K. Korhonen:** research design, interpretation, funding, manuscript revision. **Martin Englund:** research design, interpretation, manuscript revision. **Petri Tanska:** research design, analysis, interpretation, funding, manuscript revision. All authors have read and approved the final submitted manuscript.

## Conflicts of Interest

Martin Englund reports consultancy for Grünenthal Sweden AB and Key2Compliance AB.

## Supporting information

honkapaa_Supplementary_Material.
