# Supplementary material for: Depth‐Wise Changes in Tibial and Femoral Human Knee Joint Cartilage at Different Severities of Osteoarthritis
Source: J Orthop Res. 2025 Dec 13;44(1):e70104. doi: 10.1002/jor.70104 (PMC12701628; doi:10.1002/jor.70104)
Supplement: Supplementary file 1 — honkapaa_Supplementary_Material. [file JOR-44-0-s001.docx]

**Supplementary Material** **for**

**Depth-wise changes in tibial and femoral human knee joint cartilage at different severities of osteoarthritis**

*Katja Honkapää^1^, Atte S.A. Eskelinen^1^, Santtu Mikkonen^1,2^, Mohammadhossein Ebrahimi^1^, Mikko A.J. Finnilä^3,4^, Simo Saarakkala^3,4,5^, Rami K. Korhonen^1^, Martin Englund^6^, and Petri Tanska^1^

^1^ Department of Technical Physics, University of Eastern Finland, Kuopio, Finland

^2^ Department of Environmental and Biological Sciences, University of Eastern Finland, Kuopio, Finland

^3^ Research Unit of Health Sciences and Technology, Faculty of Medicine, University of Oulu, Oulu, Finland

^4^ Biocenter Oulu, University of Oulu, Oulu, Finland

^5^ Department of Diagnostic Radiology, Oulu University Hospital, Oulu, Finland

^6^ Faculty of Medicine, Department of Clinical Sciences Lund, Orthopaedics, Clinical Epidemiology Unit, Lund University, Lund, Sweden

*: Corresponding author

E-mail: [katja.honkapaa@uef.fi](mailto:katja.honkapaa@uef.fi), [petri.tanska@uef.fi](mailto:petri.tanska@uef.fi)

Telephone: +358 443408588

Address: University of Eastern Finland, Yliopistonranta 8, P.O. Box 1627, 70211, Kuopio, Finland.

*Compartment-wise analysis*

As sensitivity analysis, we checked whether inclusion of compartment (medial/lateral) to the linear mixed effects model as a fixed effect would substantially impact the model estimates. In the model, the compartment and the interaction of the OARSI group and the normalized cartilage depth were considered fixed effects, and the subject was accounted with a random intercept and variance components structure. The normalized depth was modeled with a cubic spline with a maximum of three knots. For the deep zone averages (ROI 2 in Fig. 1), we used otherwise similar linear mixed model but the compartment and the OARSI group were considered as fixed effects.

The statistical significance of compartment as a fixed effect for each parameter is shown in Supplementary Table S1. In tibia, the proteoglycan content and parallelism index estimates were higher in medial than in lateral cartilage. In femur, the proteoglycan content estimate was higher in medial than in lateral cartilage. On the other hand, collagen content, orientation angle, and anisotropy estimates were lower in medial than in lateral femoral cartilage. Although the effect of compartment was strong in general, we did not account for it in our analysis because of our limited sample size. The effect of compartment was not significant in the deep zone analysis.

**Supplementary Table S1.** Statistical significance of compartment as a fixed effect in the linear mixed model for different parameters. Bolded values represent statistical significance. ROI 2 = region of interest in the deep zone.

|  | Tibia  (depth-wise) | Femur  (depth-wise) | Tibia  (ROI 2) | Femur  (ROI 2) |
| --- | --- | --- | --- | --- |
| Proteoglycan content | ***p* = 0.0166** | ***p* = 0.00238** | *p* = 0.413 | *p* = 0.988 |
| Collagen content | *p* = 0.568 | ***p* = 0.00156** | *p* = 0.0651 | *p* = 0.623 |
| Collagen orientation angle | *p* = 0.257 | ***p* = 3.78e-10** | *p* = 0.692 | *p* = 0.447 |
| Parallelism index | ***p* < 2e-16** | ***p* = 1.05e-09** | *p* = 0.658 | *p* = 0.575 |
